# Successful pregnancy in a female with embryonal rhabdomyosarcoma of the cervix who received biopsy and chemotherapy alone without recurrence after 16 years: a case report and literature review

**DOI:** 10.1186/s12905-023-02623-6

**Published:** 2023-09-01

**Authors:** Xiuzhang Yu, Mingrong Qie, Liyan Huang, Minmin Hou

**Affiliations:** 1 Department of Obstetrics and Gynecology, West China Second University Hospital, Sichuan University, Number 20, 3rd section, South Renmin Road, Chengdu, 610041 Sichuan China; 2 Key Laboratory of Birth Defects and Related Diseases of Women and Children (Sichuan University), Ministry of Education, Number 17, 3rd section, South Renmin Road, Chengdu, 610041 Sichuan China; 3 Department of Pathology, West China Second University Hospital, Sichuan University, Number 20, 3rd section, South Renmin Road, Chengdu, 610041 Sichuan China

**Keywords:** Embryonal rhabdomyosarcoma, Organ-preserving, Chemotherapy, Pregnancy outcome

## Abstract

**Background:**

Embryonal rhabdomyosarcoma (ERMS) of the uterine cervix is rare, but the population affected is mostly underage females. The scope of surgery has now evolved from extensive to limited, and organ-preserving surgery combined with chemotherapy is recommended to preserve the patient’s fertility. However, reports of birth outcomes are rare.

**Case:**

A minor woman with cervical ERMS who underwent only an outpatient biopsy of the lesion had no residual lesion on subsequent multipoint cervical biopsy and refused radical surgery or cervical conization, after which the patient received a nonclassical regimen of chemotherapy. The patient stopped the chemotherapy on her own, but the patient conceived spontaneously 16 years later with a good pregnancy outcome and no recurrence.

**Conclusions:**

This case suggests that preservation of reproductive function is often feasible in immature women with cervical ERMS, and the prognosis is usually good as long as the primary tumour can be surgically removed and the lesion is free of residual disease. We also look forward to reports of subsequent growth and pregnancy outcomes in other children with reproductive tract RMS. In cervical ERMS, accurate evaluation of the disease and development of an individualized treatment plan are crucial, and the protection of reproductive function and psychological well-being deserves special attention.

## Introduction

According to the 2020 WHO classification of soft tissue tumours, rhabdomyosarcoma (RMS) is classified into four main subtypes: embryonal rhabdomyosarcoma (ERMS), alveolar rhabdomyosarcoma (ARMS), spindle cell/sclerosing rhabdomyosarcoma, and pleomorphic rhabdomyosarcoma. Of these, ERMS is the most common type. However, RMS of the uterine cervix is rare, and only 0.5% of primary RMSs are located in the cervix [1]; thus, the diagnosis and treatment experience of cervical RMS is very short and mainly comes from case reports, four studies by the Intergroup Rhabdomyosarcoma Study Group (IRSG): IRS-I (1972–1978), IRS-I (1978–1984), IRS-II (1984–1991) and IRS-III (1991–1997)) [2], relevant clinical studies by the International Society of Paediatric Oncology (SIOP) or other organizations, and the expert consensus from the International Soft Tissue Sarcoma Consortium (INSTRuCT) [3]. Cervical RMS usually occurs in the first two decades of life. It is important to preserve fertility to have a successful birth. However, the pregnancy outcomes of cervical RMS patients undergoing fertility-sparing surgery are rarely reported. Here, we report a case of a minor female with cervical ERMS (cervical sarcoma botryoides) who underwent only biopsy without curative surgery and received nonclassical regimen chemotherapy. She conceived spontaneously and delivered successfully without recurrence 16 years later. Combined with this particular case, we review previous reports and propose some new thoughts on the diagnosis, treatment, and prognosis of cervical ERMS. The reproductive outcomes of cervical ERMS patients are promising.

## Case

**Fig. 1:**
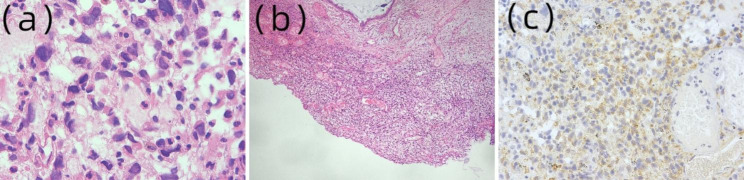
Pathologic characteristics. (a) Immature, round to spindle rhabdomyoblasts interspersed in the stroma (HE stain, ×400). (b) The tumor was growing in the cervical stroma (HE stain, ×40). (c) Immunohistochemistry showed the tumor cells to be positive for desmin (×200)

The patient was first seen in December 2005 and was 16 years old. She presented to the clinic with “vaginal discharge for six months, aggravated for one month, and prolapsed tissue from the vagina that occurred twice.“ Three months prior, the patient felt a fleshy, bright red tissue, which was approximately 1 cm in size, prolapsing from her vagina when she urinated. At that time, the patient was advised to undergo surgery. However, she refused, and the tissue later self-retracted into the vagina. The tissue prolapsed outside the vagina again one month later, so the patient underwent a biopsy in our outpatient clinic for the tissue outside the vaginal orifice (without disrupting the hymen). Immunohistochemistry revealed desmin (++), myoglobin (-), myogenin (-), and myoD1 (-). In conjunction with the microscopic presentation, this patient was diagnosed with cervical sarcoma botryoides (Fig. 1). After the patient was hospitalized, at the request of the patient’s family, we performed a cervical examination before deciding on the surgical procedure. A speculum was placed after general anaesthesia was induced, and we found that the vagina was patent with no tissue inside. The cervical area was slightly bulgy at points 7–9 but without any polyp-like tissue. Fractional curettage and multipoint cervical biopsy were performed, which showed no residual tumour. An intraoperative review of the outpatient biopsy section confirmed the diagnosis of cervical sarcoma botryoides. The tumour was classified as stage I according to the intergroup rhabdomyosarcoma study criteria and TXN0M0 according to the tumour node metastasis staging system. The patient’s family refused extensive hysterectomy and pelvic lymph node dissection, and the operation ended. The patient received chemotherapy with the IEP (etoposide, cisplatin, ifosfamide) regimen for four cycles (initially planned for six cycles; after four cycles, the patient stopped chemotherapy on her own) without any other treatment. Thereafter, the patient did not return to the hospital for regular follow-up until 2021, when she was seen again in our hospital for pregnancy. The patient eventually delivered successfully in 2022 and has had no signs of recurrence to date. The newborn had no abnormalities. Patient consent was obtained for this case report.

## Discussion and conclusions

The clinical presentation of cervical RMS is mostly a cervical mass and/or vaginal bleeding. Typical tumours are nodular, papillary, polypoid or grape-like masses, but they may grow infiltratively, involve surrounding tissues or metastasize distantly. An MRI scan of the abdominopelvic region is preferred as an adjunctive test. Cystoscopy, vaginoscopy, and bimanual examination (or rectoscopy if necessary) are also recommended [3]. Before 1972, the majority of children with uterine RMS were treated with radical surgery. Starting in 1976, the IRSG used initial surgery followed by chemotherapy for cervical polyp-like lesions. This patient had cervical ERMS of the botryoid subtype, which was called cervical sarcoma botryoides at that time. The particular features of this case were as follows: (1) the patient did not undergo curative surgery, whether polypectomy, hysterectomy or others, and only the biopsy was performed without destruction of the hymen; (2) the patient’s chemotherapy regimen was not classical vincristine, actinomycin-D, cyclophosphamide (VAC) or vincristine, ifosfamide, actinomycin (VIA), and the chemotherapy cycles were inadequate. However, there were no signs of recurrence; and (3) the patient conceived spontaneously and had a good pregnancy outcome.

The aetiology of ERMS is unclear. Recent studies have found that there may be a correlation between pathogenic variants of DICER1 and ERMS, especially cervical EMRS [4, 5]. Unfortunately, genetic testing was not performed on this patient.

The previous view of the primary surgical approach was extensive hysterectomy with adequate vaginal excision and simultaneous pelvic and para-aortic lymph node dissection. However, patients with cervical ERMS are usually very young. The inability to have children or even normal development of female sexual characteristics after radical surgery causes substantial physical and psychological harm. The scope of surgery has now evolved from extensive to limited [6]. The IRSG recommends that less vigorous operative resection may be possible in combination with primary chemotherapy when treating uterine RMS [7]. INSTRuCT suggests more conservative approaches with chemotherapy alone in patients with complete response or organ-sparing surgery in combination with intracavitary brachytherapy or external beam radiotherapy. All children with uterine ERMS should be considered for fertility preservation, but hysterectomy is recommended for children with persistent corpus uteri tumours [3]. Surgery should be performed to remove the primary tumour and some normal tissues around its periphery to achieve lesion-free margins, followed by chemotherapy. Surgical options include simple mass excision, cervical conization, radical hysterectomy, etc. The exact procedure and scope of surgery should be determined by the patient’s age, size of the lesion, tissue type, and whether it infiltrates the surrounding organs. However, recurrence after conservative treatment is not uncommon, especially in patients who have not received postoperative chemotherapy or have received insufficient cycles of chemotherapy [8]. RMS is a chemosensitive tumour. Even for IRSG Group I (localized disease, completely excised, no microscopic residual tumour) patients, postoperative chemotherapy is recommended. In Europe, the standard chemotherapy regimen for ERMS is VIA, while IRSG recommends VAC [9]. If patients with tumours localized at the cervix demonstrate an incomplete response after induction chemotherapy, brachytherapy should be carried out [3]. The recently reported 10-year overall survival rate was 92% for vaginal and uterine RMS in children and adolescents, and approximately half of these patients did not undergo radical surgery [10]. The presence of residual lesions after the initial surgery is the most important prognostic factor. Factors associated with prognosis include disease stage, age, tissue subtype, regional lymph node involvement, distant metastasis, and treatment modality [6]. It is currently considered that well-defined (polypoid) presentations, embryonal histology, and superficial tumours are indications for preserving reproductive function, while alveolar/pleomorphic histology and deeply invasive disease increase the risk of tumour recurrence [9]. In this case, the patient underwent an outpatient biopsy of the vaginal orifice tissue without disruption of the hymen and then underwent only fractional curettage and multipoint cervical biopsy without any curative surgery during her hospitalization. She then received an inadequate course of chemotherapy. To date, it has been 16 years without recurrence. This case provides further clinical thoughts on the scope of surgery for local cervical ERMS. For early lesions, is mass excision alone sufficient as long as the margins are negative? Is cervical conization or a more extensive hysterectomy necessary? Our patient received the IEP regimen, not the commonly used chemotherapy regimen in the previous guidelines, which has been reported in some other sites of rhabdomyosarcoma, but the patient also experienced a good curative effect. The overall prognosis for cervical RMS in immature patients is good, and conservative surgical resection combined with chemotherapy is recommended. These research results suggest that fertility-sparing surgery combined with chemotherapy is recommended in immature cervical RMS patients.

As mentioned earlier, the current treatment philosophy for ERMS of the genital tract is to protect the patient’s reproductive function as much as possible. However, pregnancy outcomes after treatment were less frequently reported. Two patients with cervical mass excision who were found to have ERMS during pregnancy were reported. They both underwent radical surgery during or after caesarean delivery in late pregnancy, and one of them had chemotherapy with a VAC regimen during pregnancy [11, 12]. Another case reported a patient with an 8 × 7 cm cervical ERMS lesion who had an unplanned pregnancy after 3 cycles of doxorubicin and ifosfamide. She was untreated during pregnancy and successfully delivered a normal baby. After 6 months, a local polypoid area less than 1 cm in diameter was found, and conization and pelvic lymphadenectomy were performed [13]. Even without radiotherapy, high doses of alkylating agents, such as cyclophosphamide and ifosfamide, are considered associated with decreased germ cell function and fertility [3]. The latest consensus indicated that 37% of women could become pregnant after cervical cancer surgery, and the live birth rate was 67% [3]. The patient in this case had a successful spontaneous pregnancy 16 years after her diagnosis of cervical ERMS without recurrence. It also makes us look forward to reports on the subsequent growth and pregnancy outcomes of other children with genital tract RMS.

In recent years, the treatment concept has changed from extensive surgical resection to limited surgery combined with chemotherapy and radiotherapy, but the prognosis has improved dramatically. This case suggests that fertility-sparing surgery could be the first choice for immature patients with embryonal rhabdomyosarcoma of the uterine cervix, and the prognosis is usually good as long as the primary tumour can be surgically removed and the lesion is free of residual disease. Pregnancy outcomes for these patients are also promising. As far as we know, there are very few reports on the pregnancy outcomes of patients with cervical ERMS after fertility preservation surgery, so our report is of great importance for the treatment concept of cervical ERMS. The limitation of this study is that it is only a single case report, and future follow-up of other minor cervical ERMS patients will be needed to provide stronger evidence for the above conclusion. It is essential to emphasize that chemotherapy is significant in reducing recurrence. For paediatric patients with genital tract tumours, protection of reproductive function and psychological health is of particular importance. The issues of how to accurately assess the patient’s condition, grasping the surgical protocols and developing the chemoradiotherapy plan deserve further exploration.

## Data Availability

The data obtained during the current study are available from the corresponding author on reasonable request.

## Abbreviations

RMS: Rhabdomyosarcoma
ERMS: embryonal rhabdomyosarcoma, ARMS:alveolar rhabdomyosarcoma
SIOP: the International Society of Pediatric Oncology
INSTRuCT: the International Soft Tissue Sarcoma Consortium
